# Matrine Attenuates Neurological Deficits and Neuroinflammation by Inhibiting the HMGB1/RAGE Axis and Ferroptosis in Intracerebral Hemorrhage Mice

**DOI:** 10.1002/kjm2.70157

**Published:** 2025-12-16

**Authors:** Pan‐Di Chen, Tao‐Tao Shi, Dan‐Yang Hu, Xiao‐Na Su, Jian‐Xiang Jin, Mao‐Song Chen, Hao Wang, Wei Chen

**Affiliations:** ^1^ Department of Neurosurgery The Affiliated Lihuili Hospital of Ningbo University Ninbo China

**Keywords:** ferroptosis, intracerebral hemorrhage, matrine, neuroinflammation, RAGE

## Abstract

Intracerebral hemorrhage (ICH) is a life‐threatening stroke subtype that lacks effective drug therapy. In the context of ICH, neuroinflammation, and ferroptosis are key contributors to secondary brain injury. In this study, we assessed the neuroprotective effects of matrine (MAT) and its mechanism of action involving the HMGB1/RAGE axis in an ICH mouse model. ICH was induced in mice by autologous blood injection. MAT (1.5 or 20 mg/kg) was administered orally 5 min postinduction. ICH significantly upregulated RAGE and its associated proteins (HMGB1, protein kinase C [PKC], phospholipase C [PLC], and β‐amyloid), confirming pathway activation. Molecular docking revealed a specific hydrogen bond interaction between MAT and RAGE but not with PKC or PLC. MAT improved neurological performance, reduced hematoma volume and brain edema, and restored blood–brain barrier integrity through upregulation of tight junction proteins (ZO‐1, Occludin, Claudin‐4). MAT suppressed activation of the HMGB1/RAGE pathway and downstream kinases JNK, p38, ERK1/2, and NF‐κB p65. It also decreased IL‐1β, IL‐6, and TNF‐α levels, reduced Bax, increased Bcl‐2, and mitigated neuronal apoptosis. Additionally, MAT lowered Fe^2+^ accumulation, inhibited ACSL4, and restored GPX4 levels, indicating ferroptosis suppression. RAGE knockdown mimicked the effects of MAT and negated its therapeutic benefits. MAT induced no detectable liver or kidney toxicity. This study is the first to demonstrate that MAT alleviates ICH‐induced neurological deficits by targeting the HMGB1/RAGE axis‐mediated ferroptosis, thereby extending the current understanding of the neuroprotective mechanisms of MAT beyond its known anti‐inflammatory and antioxidant actions.

## 1 Introduction

Intracerebral hemorrhage (ICH) is a devastating form of stroke, accounting for 10%–15% of all stroke cases, and disproportionately contributes to stroke‐related mortality and disability [1]. Despite advances in surgical interventions and intensive care, effective pharmacological treatments for ICH remain unavailable [2]. Secondary brain injuries, including oxidative stress, neuroinflammation, blood–brain barrier (BBB) disruption, and programmed cell death, play a central role in neurological deterioration following ICH [3, 4, 5]. Among the emerging forms of programmed cell death, ferroptosis, characterized by iron accumulation and lipid peroxidation, has gained attention as a critical mechanism underlying neuronal damage in ICH [6]. Hence, targeting and suppressing ferroptosis has shown potential for reducing brain injury in preclinical ICH models [7]. For example, both dauricine and withaferin A have been proven to protect against brain injury through repressing ferroptosis in the mouse model of ICH [8, 9]. More recently, cinnamaldehyde, a bioactive component of the traditional Chinese medicine (TCM) *Cinnamomum cassia* , was shown to be remarkably effective in repairing ICH‐induced brain damage via its antiferroptotic and anti‐inflammatory properties [10].

A growing body of evidence indicates that the high‐mobility group box 1 (HMGB1)/receptor for advanced glycation end‐products (RAGE) signaling axis plays a central role in mediating neuroinflammation and neuronal death following ICH [11, 12]. Activation of this pathway has been linked to increased oxidative stress, proinflammatory cytokine production, and BBB breakdown. RAGE signaling has also been shown to influence pathways associated with ferroptosis regulation. The binding of AGEs to RAGE increases reactive oxygen species (ROS) generation and induces oxidative stress‐induced lipid peroxidation, thereby leading to increased ferroptosis‐dependent cytotoxicity [13]. However, therapeutic agents that concurrently target the HMGB1/RAGE axis and ferroptosis are yet to be explored.

Matrine (MAT), a natural quinolizidine alkaloid isolated from the TCM *Sophora flavescens* , has shown anti‐inflammatory, antioxidant, and antiapoptotic properties in various neurological disease models, including ischemic stroke and traumatic brain injury [14, 15]. Similar to many TCM‐derived agents, MAT is characterized by multitarget pharmacological activity and a favorable safety profile [16, 17]. However, its therapeutic efficacy in ICH and the underlying mechanisms remain largely undefined.

Considering the complex pathophysiology of ICH and the multimodal actions of MAT, in the present study, we incorporated molecular docking analysis to predict potential interactions between MAT and key proteins within the RAGE signaling pathway, thereby establishing a theoretical basis for subsequent biological validation. The neuroprotective efficacy of MAT was systematically evaluated in a murine model of ICH, with mechanistic investigations focusing on its regulatory effects on the HMGB1/RAGE axis and ferroptosis‐related processes. Collectively, these findings provide experimental evidence for the therapeutic relevance of MAT in ICH and broaden the pharmacological understanding of TCM‐derived compounds in hemorrhagic stroke.

## 2 Material and Methods

### 2.1 Ethics Statement

All animal experiments were approved by the Institutional Animal Care and Use Committee of the Zhejiang Huitong Test & Evaluation Technology Group Co. Ltd. (Approval No. HT‐2025‐LWFB‐0045) and were conducted in accordance with the National Institutes of Health Guide for the Care and Use of Laboratory Animals.

### 2.2 Animals and Experimental Design

Male wild‐type (WT) C57BL/6J mice and *Rage* knockout (*Rage* KO) mice (8–10 weeks old, 22–25 g) were housed in a specific‐pathogen‐free facility under controlled temperature (22°C ± 2°C), humidity (55% ± 5%), and a 12‐h light/dark cycle, with food and water available ad libitum. After 7 days of acclimatization, WT mice were randomly assigned into five experimental groups (*n* = 6–12 per group): Sham (*n* = 12), ICH (*n* = 12), ICH + MAT (1 mg/kg) (*n* = 6), ICH + MAT (5 mg/kg) (*n* = 6), and ICH + MAT (20 mg/kg) (*n* = 12). *Rage* KO mice were randomly divided into three groups: Sham, ICH, and ICH + MAT (20 mg/kg). Group assignments and all assessments were performed by investigators who were blinded to the treatment using coded animal IDs.

### 2.3 ICH Induction

ICH was induced via stereotactic injection of autologous blood [18]. Briefly, mice were anesthetized with an intraperitoneal injection of sodium pentobarbital (50 mg/kg). Once anesthesia was confirmed, the mice were fixed in a stereotaxic apparatus (RWD Instruments, China). A burr hole was drilled 0.2 mm anterior and 2.2 mm lateral to the bregma, and a 26‐gauge Hamilton syringe was inserted to a depth of 3.5 mm from the dura. Subsequently, 30 μL of autologous blood was injected into the right basal ganglia over 15 min (2 μL/min). The needle was left in place for 10 min after injection to prevent reflux before being slowly withdrawn. The burr hole was sealed with bone wax, and the incision was sutured. Sham‐operated mice were subjected to needle insertion without blood injection.

### 2.4 Drug Administration

MAT (purity ≥ 98%, Sigma) was dissolved in sterile saline and administered by oral gavage at doses of 1, 5, or 20 mg/kg, starting 5 min after ICH induction and continued once daily until the designated time point. The gavage volume was standardized to 10 mL/kg. The control group received an equal volume of vehicle (saline).

### 2.5 Neurological Function Assessment

Neurological function was evaluated using the modified neurological severity score (mNSS) [19] and the rotarod test [20]. The mNSS is a composite scoring system that incorporates motor, sensory, reflex, and balance assessments. Scores range from 0 to 18, where 0 represents normal function and 18 indicates the most severe neurological deficit. Each failed task or missing reflex contributed 1 point, with severity categorized as mild (1–6), moderate (7–12), or severe (13–18) impairment. The assessments were independently conducted by two trained investigators who were blinded to the treatment groups. mNSS evaluations were performed at baseline (presurgery) and 1, 3, 7, and 14 days after ICH induction.

For the rotarod test, mice were pretrained for three consecutive days at 10 rpm using a rotarod apparatus (ZH‐YLS‐4C, Zhenghua Instruments, China), and the performance was recorded as the latency to fall across three averaged trials per time point. During testing, each mouse underwent three trials at 5‐min intervals. The average latency to fall was also recorded.

### 2.6 Hematoma Volume

At 72 h post‐ICH, the mice were euthanized, and their brains were rapidly removed. To measure the hematoma volume, the brains were sliced into 1‐mm coronal sections and imaged. The hematoma area was quantified using ImageJ software, and the total volume was calculated by integration.

### 2.7 BBB Permeability

Evans blue (EB) extravasation was used to evaluate BBB integrity. Mice were administered a 2% EB solution (4 mL/kg) via the tail vein. After a 3‐h circulation period, the animals were deeply anesthetized using sodium pentobarbital (50 mg/kg, i.p.) and perfused transcardially with 200 mL of cold phosphate‐buffered saline to eliminate intravascular dye. The brains were perfused with saline, weighed, and homogenized in formamide (1 mL/100 mg). After incubation at 55°C for 24 h, the samples were centrifuged, and the absorbance of the supernatant was measured at 620 nm.

### 2.8 Histology and Nissl Staining

Formalin‐fixed paraffin‐embedded brain sections (5 μm) were stained with hematoxylin and eosin (H&E) to evaluate the general histopathology. Nissl staining was performed using a 0.1% cresyl violet solution to assess neuronal survival. Images were captured using a light microscope (Olympus BX53, Japan).

### 2.9 Enzyme‐Linked Immunosorbent Assay (ELISA)

Perihematomal brain tissue was homogenized and centrifuged to obtain the supernatants. Commercial ELISA kits were used to quantify the levels of IL‐1β (Invitrogen; Cat.# 88‐7013A‐88), IL‐6 (Invitrogen; Cat.# 88‐7064‐88), and TNF‐α (Invitrogen; Cat.# 88‐7324‐88) according to the manufacturer's instructions.

### 2.10 Western Blot Analysis

For western blot analysis, protein samples were extracted from the perihematomal brain tissue surrounding the hematoma in the ipsilateral hemisphere. Proteins were extracted using RIPA lysis buffer containing protease and phosphatase inhibitors. Equal amounts of protein (30 μg) were separated by SDS‐PAGE and transferred to PVDF membranes. After blocking, membranes were incubated overnight at 4°C with primary antibodies against RAGE (MyBioSource; Cat.# MBS9601481; 1:1000), β‐amyloid (Aβ1–42; Cell Signaling; clone D3E10; Cat.# 12843S; 1:1000), HMGB1 (Cell Signaling; Cat.# 3935S; 1:1000), protein kinase C (PKC; Proteintech; Cat.# 12919‐1‐AP; 1:1000), and phospholipase C (PLC; Cell Signaling; Cat.# 2822S; 1:1000), ERK1/2 (Cell Signaling; Cat.# 9102S; 1:1000), p‐ERK1/2 (Cell Signaling; Cat.# 4370S; 1:1000), NF‐κB p65 (Invitrogen; Cat.# 51‐0500; 1:1000), p‐NF‐κB p65 (Invitrogen; Cat.# MA5‐15160; 1:1000), GPX4 (Invitrogen; Cat.# JU11‐31; 1:500), ACSL4 (Cell Signaling; Cat.# 38493S; 1:1000), Bax (Invitrogen; Cat.# MA5‐35342; 1:1000), Bcl‐2 (Cell Signaling; Cat.# 3498S; 1:1000), ZO‐1 (Cell Signaling; Cat.# 5406S; 1:1000), Occludin (Cell Signaling; Cat.# 91131S; 1:1000), Claudin‐4 (Abcam; Cat.# ab210796; 1:1000), and β‐actin (loading control; Cell Signaling; Cat.# 4970S; 1:5000). HRP‐conjugated secondary antibodies (Cell Signaling Cat.# 7074S; 1:5000) were used, and detection was performed with ECL chemiluminescence. Band intensities were analyzed using ImageJ software.

### 2.11 Molecular Docking

The 3D structure of MAT was obtained from PubChem, and target protein structures (RAGE, PKC, and PLC) were obtained from the Protein Data Bank. The ligand and protein preparations were performed using AutoDockTools 1.5.6. Docking was conducted using AutoDock Vina, and the binding site was defined based on known active sites. The results were ranked by binding energy, and hydrogen‐bond interactions were visualized using PyMOL and Discovery Studio Visualizer [21].

### 2.12 Liver and Kidney Toxicity Evaluation

The liver and kidney tissues were processed for H&E staining to evaluate toxicity. Serum samples were collected for biochemical analysis of ALT, AST, BUN, and creatinine using commercial kits purchased from MyBioSource (ALT, Cat.# MBS3805312; AST, Cat.# MBS3805769; BUN, Cat.# MBS2611085; creatinine, Cat.# MBS2611108).

### 2.13 Statistical Analysis

Data are presented as the mean ± standard deviation (SD). Statistical analyses were performed using GraphPad Prism 9.0. Multiple groups were compared using one‐ or two‐way analysis of variance followed by Tukey's post hoc test. Statistical significance was set at *p* < 0.05.

## 3 Results

### 3.1 RAGE Axis Is Activated Following ICH Induction in Mice

**FIGURE 1:**
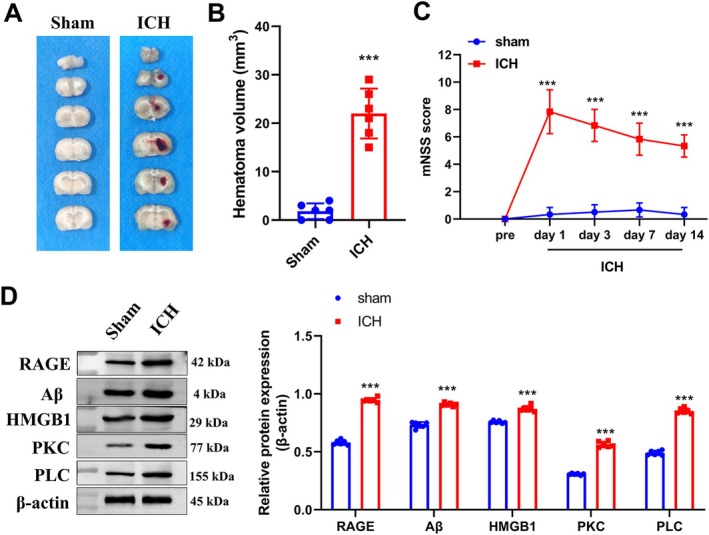
Activation of RAGE‐related signaling following ICH. (A) Representative coronal brain sections showing hematoma formation in the ICH group versus the sham group at 72 h postsurgery. (B) Quantification of hematoma volume (mm^3^) revealed a significant increase in ICH mice. (C) mNSS assessed at baseline and at 1, 3, 7, and 14 days after ICH. Neurological deficits were significantly elevated in the ICH group at all time points postinjury. (D) Western blot analysis of RAGE and related pathway proteins, including Aβ, HMGB1, PKC, and PLC. Densitometric analysis showed significantly increased protein expression levels in ICH mice. Data are presented as the mean ± SD (*n* = 6 per group). ***p < 0.001 vs. sham.

To confirm the successful establishment of the ICH model and investigate the involvement of the RAGE pathway, histological, behavioral, and molecular analyses were conducted at early time points postinjury. Representative coronal brain sections showed obvious hematoma formation in the ICH group compared with the sham group (Figure 1A). Quantitative analysis confirmed a significant increase in hematoma volume in ICH mice (Figure 1B, ***p < 0.001). Correspondingly, the mNSS was markedly elevated on days 1, 3, 7, and 14 post‐ICH, indicating sustained neurological deficits (Figure 1C, ***p < 0.001).

Western blot analysis revealed that the expression levels of RAGE and its related downstream proteins were significantly upregulated in perihematomal tissues following ICH. Specifically, protein levels of RAGE, amyloid‐β (Aβ), HMGB1, protein kinase C (PKC), and phospholipase C (PLC) were all increased in the ICH group compared with sham controls (Figure 1D, ***p < 0.001 for all). These results suggest that the HMGB1/RAGE signaling axis and its downstream mediators are robustly activated during the acute phase of ICH.

### 3.2 MAT Selectively Binds to RAGE Through Hydrogen Bonding at Asp81

**FIGURE 2:**
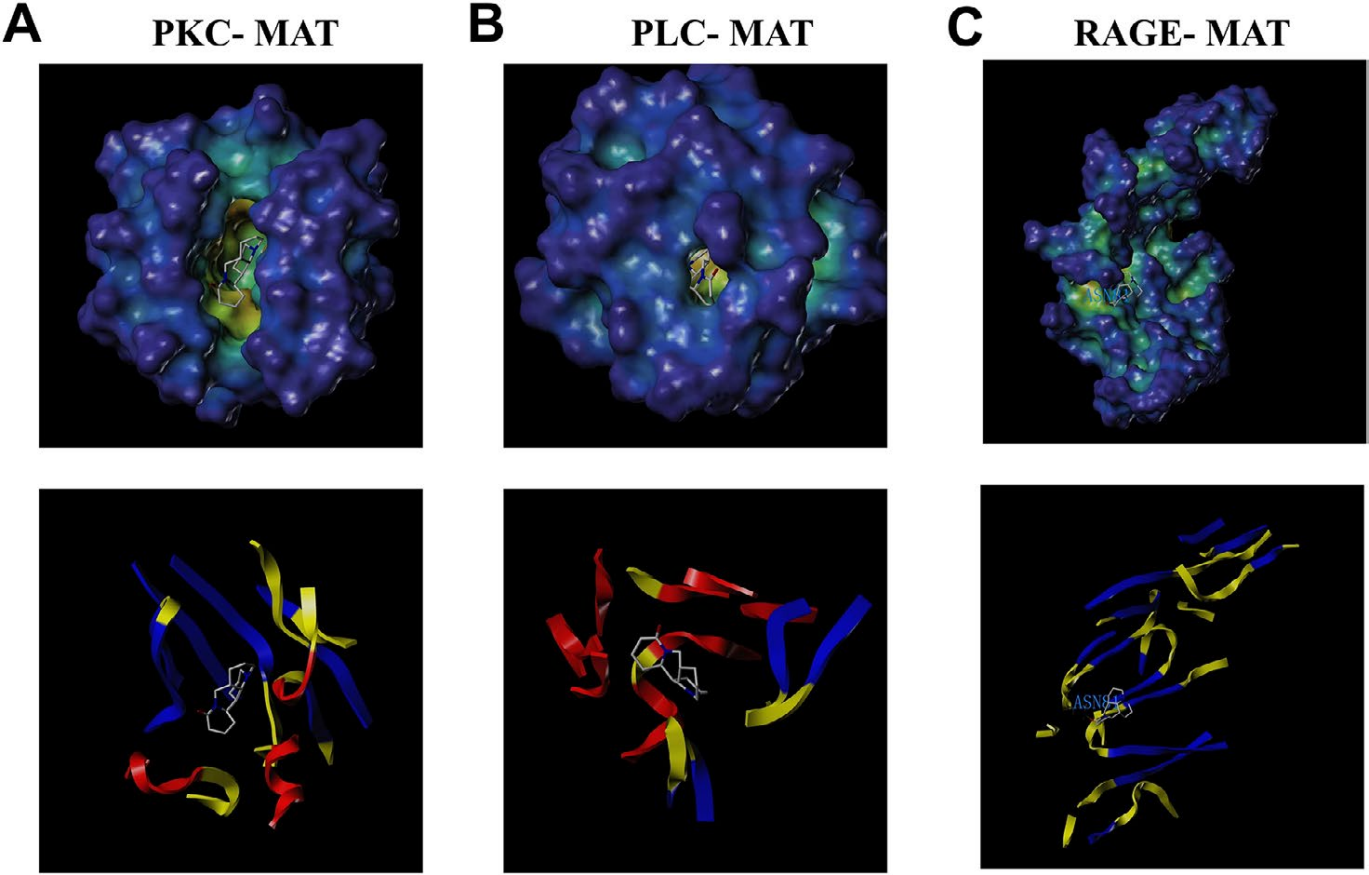
Molecular docking analysis of MAT with RAGE axis‐related proteins. Docking models showing the interaction of MAT with (A) PKC, (B) PLC, and (C) RAGE. Surface views (top panels) and ribbon structures (bottom panels) were generated to visualize the ligand‐binding pocket. Hydrogen bond formation between MAT and Asp81 of RAGE is indicated in the ribbon model, suggesting selective affinity.

To explore the potential binding interactions between MAT and the proteins involved in the RAGE signaling pathway, molecular docking simulations were conducted using AutoDock Vina. Three‐dimensional binding models revealed that MAT could enter the ligand‐binding pockets of PKC, PLC, and RAGE (Figure 2A–C). The predicted binding energies (*ΔG*) were –8.1 kcal/mol for MAT–RAGE, −4.3 kcal/mol for MAT–PKC, and −5.6 kcal/mol for MAT–PLC, indicating a markedly stronger binding affinity of MAT toward RAGE. Among these, only the MAT–RAGE complex exhibited clear hydrogen bond formation. Specifically, MAT formed a stable hydrogen bond with the aspartic acid residue at position 81 (Asp81) within RAGE, suggesting a direct molecular interaction (Figure 2C). In contrast, no hydrogen bond interactions were observed between MAT and PKC (Figure 2A) or PLC (Figure 2B). These findings imply that MAT may exert its pharmacological effects by directly targeting RAGE and subsequently inhibiting RAGE‐mediated downstream signaling cascades.

### 3.3 MAT Alleviates Neurological Deficits and BBB Disruption in ICH Mice

**FIGURE 3:**
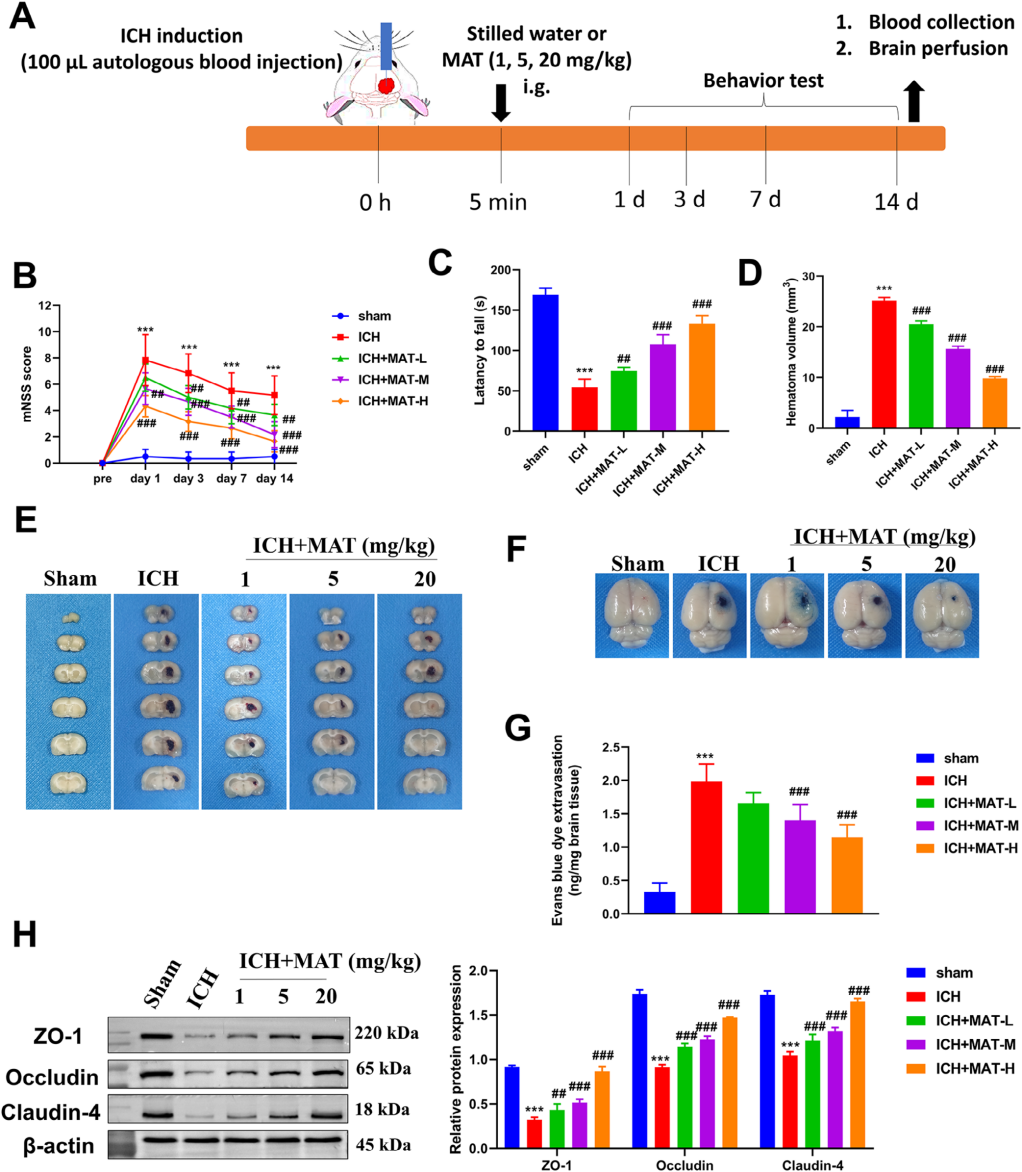
MAT improves neurological function and blood–brain barrier integrity following ICH. (A) Schematic illustration of the experimental design and timeline. (B) mNSS and (C) rotarod test results at indicated time points. (D and E) Representative coronal brain sections and quantification of hematoma volume at 72 h post‐ICH. (F and G) Evans blue extravasation images and quantitative data showing BBB permeability. (H) Western blot analysis and densitometry of tight junction proteins (ZO‐1, Occludin, and Claudin‐4) in perihematomal tissue. Data are presented as the mean ± SD (*n* = 6 per group). ***p < 0.001 vs. sham; ##*p* < 0.01, ###*p* < 0.001 vs. ICH.

To evaluate the therapeutic effect of MAT following ICH, mice were treated with different doses (1, 5, or 20 mg/kg) of MAT via oral gavage, starting 5 min postinjury (Figure 3A). Behavioral assessments revealed that ICH‐induced neurological impairment was significantly improved by MAT in a dose‐dependent manner. The mNSS scores were markedly reduced on days 1, 3, 7, and 14 in the MAT‐treated groups compared with those in the untreated ICH group (Figure 3B). Similarly, MAT significantly prolonged the latency to fall in the rotarod test, indicating improved motor coordination (Figure 3C). Consistently, MAT administration led to a significant reduction in hematoma volume (Figure 3D,E). Furthermore, the Evans blue dye extravasation assay demonstrated that MAT treatment attenuated ICH‐induced BBB leakage (Figure 3F,G). Western blot analysis of BBB‐associated tight junction proteins showed that MAT significantly upregulated the expression of ZO‐1, Occludin, and Claudin‐4 in the perihematomal regions (Figure 3H), supporting its role in BBB repair. These findings suggest that MAT confers neuroprotective effects against ICH‐induced secondary injury.

### 3.4 MAT Exerts Neuroprotective Effects by Reducing Neuronal Damage, Apoptosis, and Neuroinflammation Without Liver or Kidney Toxicity

**FIGURE 4:**
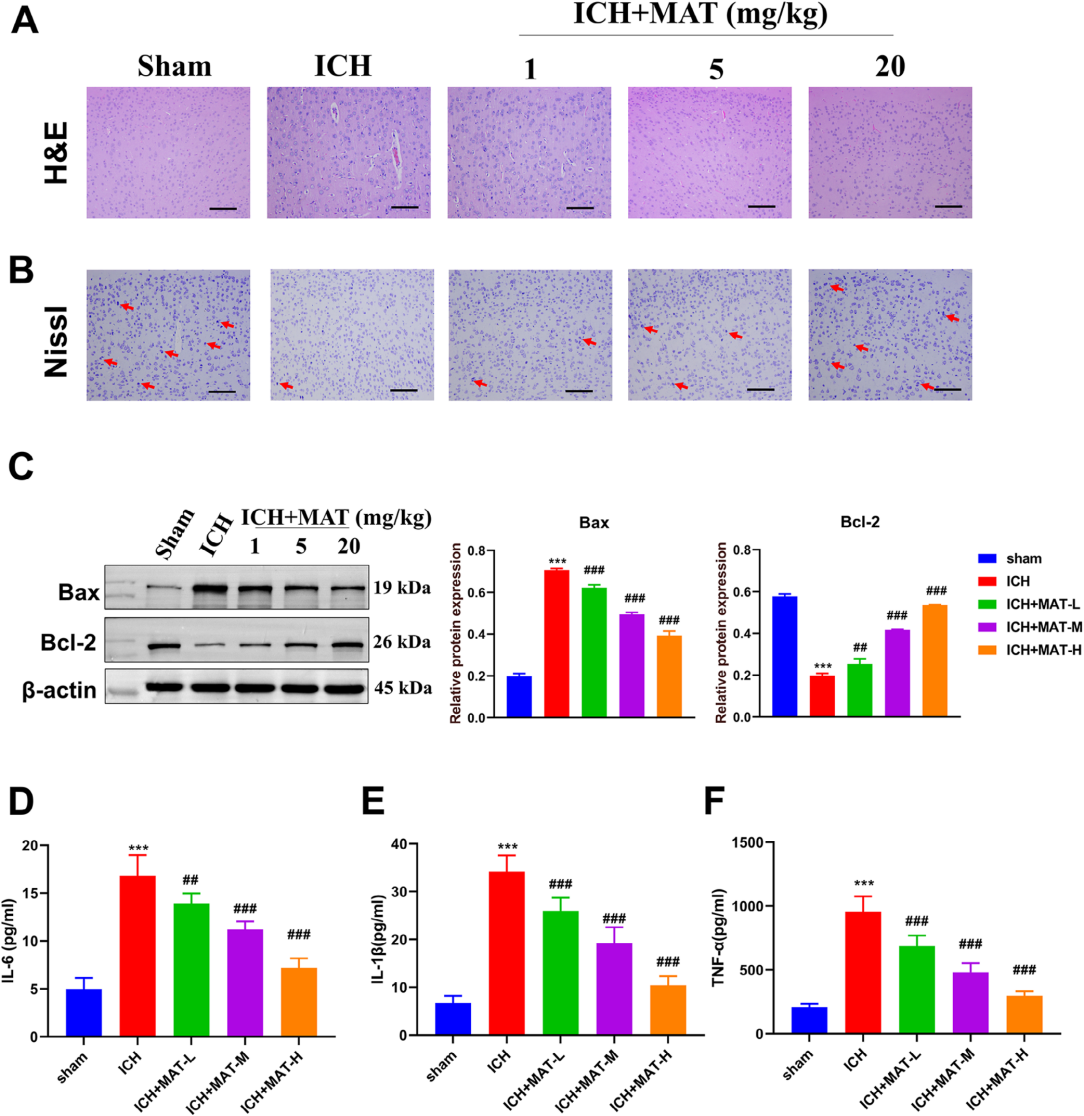
MAT reduces neuronal injury, apoptosis, and neuroinflammation following ICH. (A) Representative H&E‐stained brain sections showing tissue damage in perihematomal regions. (B) Nissl staining revealing neuronal integrity (red arrows denote surviving neurons). (C) Western blot and quantification of Bax and Bcl‐2 protein levels. (D–F) ELISA analysis of inflammatory cytokines IL‐6, IL‐1β, and TNF‐α in perihematomal brain tissue. Data are expressed as the mean ± SD (*n* = 6 per group). ***p < 0.001 vs. sham; #*p* < 0.05, ##*p* < 0.01, ###*p* < 0.001 vs. ICH.

We next performed histological and molecular analyses to investigate the neuroprotective effects of MAT against ICH. H&E staining revealed substantial neuronal degeneration, vacuolization, and tissue disorganization in the perihematomal region of the ICH mice. However, MAT treatment mitigated these morphological abnormalities in a dose‐dependent manner (Figure 4A). Nissl staining consistently showed a marked reduction in the number of intact Nissl‐positive neurons in the ICH group, which was significantly reversed by MAT administration (Figure 4B). Western blot analysis revealed increased proapoptotic Bax and decreased antiapoptotic Bcl‐2 expression in ICH mice, whereas MAT treatment downregulated Bax and upregulated Bcl‐2 expression in a dose‐dependent manner (Figure 4C). ELISA analysis further indicated that the levels of inflammatory cytokines, including IL‐6, IL‐1β, and TNF‐α, were significantly elevated in the ICH group. MAT intervention markedly suppressed the expression of these cytokines, suggesting attenuation of neuroinflammation (Figure 4D–F). Collectively, these data support the hypothesis that MAT alleviates ICH‐induced brain injury through antiapoptotic and anti‐inflammatory mechanisms.

To assess the potential systemic toxicity of MAT, the liver and kidney tissues were subjected to histopathological and biochemical analyses. H&E staining revealed no significant histological abnormalities in either the liver or kidney in the MAT‐treated groups compared with the sham and ICH groups (Figure S1A,B). Serum biochemical indices, including ALT, AST, BUN, and creatinine, remained within normal ranges across all MAT doses (1, 5, and 20 mg/kg), with no statistically significant differences observed (Figure S1C–F). These results indicate that the oral administration of MAT is well tolerated and does not cause hepatic or renal toxicity at therapeutic doses.

### 3.5 MAT Inhibits RAGE Downstream Signaling and Ferroptosis Following ICH

**FIGURE 5:**
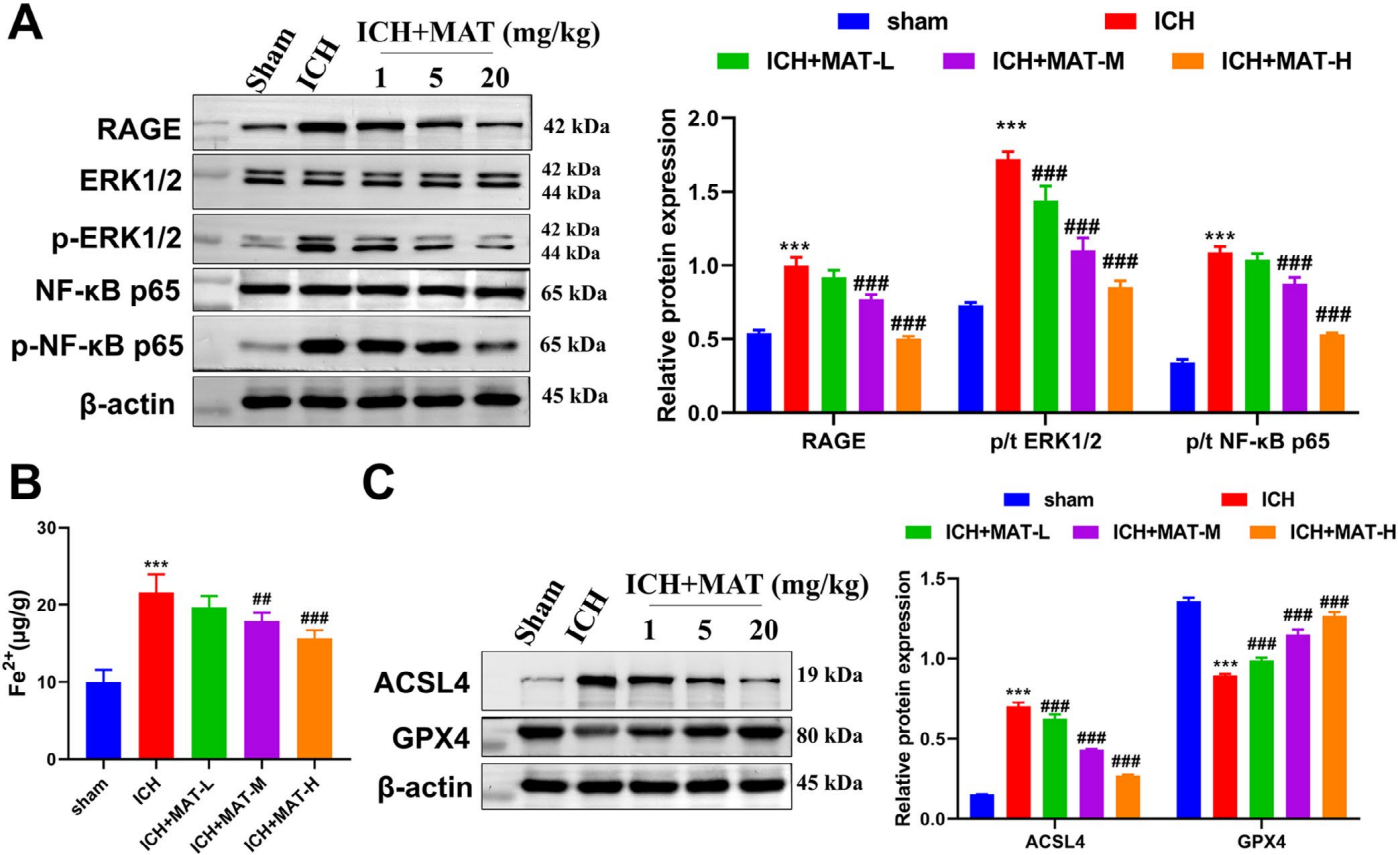
MAT suppresses RAGE pathway activation and ferroptosis after ICH. (A) Western blot analysis of RAGE, ERK1/2, p‐ERK1/2, NF‐κB p65, and p‐NF‐κB p65 in perihematomal tissue. (B) Quantification of the Fe^2+^ concentration in brain tissue. (C) Expression of the ferroptosis markers ACSL4 and GPX4 analyzed by western blot and densitometry. Data are expressed as the mean ± SD (*n* = 6 per group). ***p < 0.001 vs. sham; ##*p* < 0.01, ###*p* < 0.001 vs. ICH.

To further elucidate the mechanisms underlying the neuroprotective effects of MAT, we investigated its effect on the RAGE signaling cascade and ferroptosis markers. Western blot analysis showed a significant upregulation of RAGE, phosphorylated ERK1/2 (p‐ERK1/2), and phosphorylated NF‐κB p65 (p‐p65) in the brain tissue of ICH mice, indicating activation of the RAGE‐related inflammatory pathway (Figure 5A). Treatment with MAT dose‐dependently suppressed the expression of RAGE, as well as the phosphorylation levels of ERK1/2 and NF‐κB p65.

In addition, ICH significantly increased the level of ferrous iron (Fe^2+^) in perihematomal regions, a hallmark of ferroptosis. However, this increase was attenuated by MAT, particularly at higher doses (Figure 5B). Consistent with this finding, the ferroptosis‐related protein ACSL4 was markedly upregulated, whereas GPX4 was downregulated in ICH mice. MAT treatment reversed these alterations by decreasing ACSL4 expression and restoring GPX4 expression in a dose‐dependent manner (Figure 5C). These findings suggest that MAT partially inhibits ferroptosis by downregulating RAGE‐mediated inflammatory signaling.

### 3.6 MAT Confers Neuroprotection via a RAGE‐Dependent Mechanism

**FIGURE 6:**
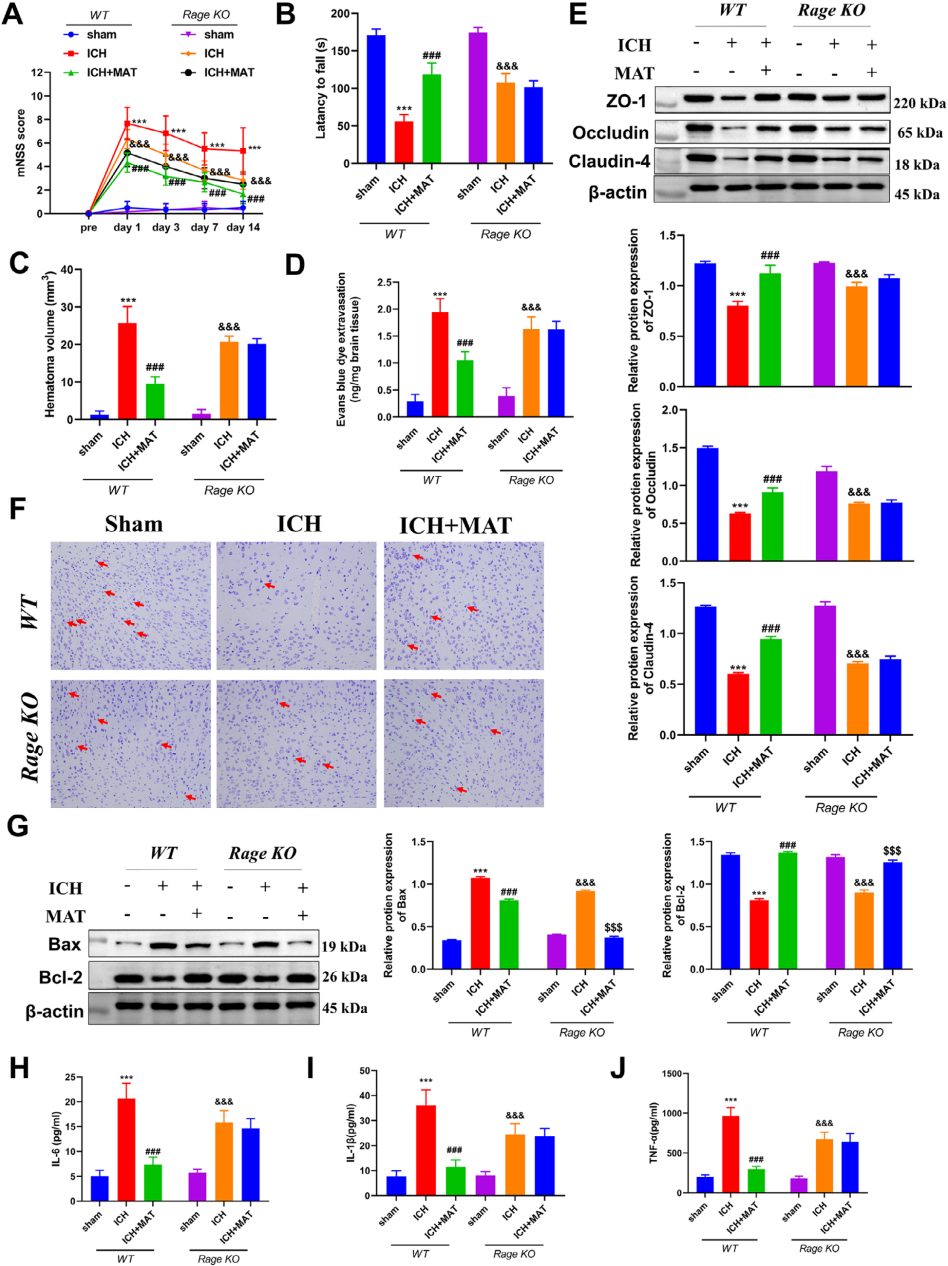
RAGE deficiency abolishes the neuroprotective effects of MAT following ICH. (A) Modified neurological severity scores and (B) rotarod latency. (C) Hematoma volume and (D) Evans blue dye extravasation. (E) Expression of the tight junction proteins ZO‐1, Occludin, and Claudin‐4. (F) Nissl‐stained brain sections indicating neuronal survival (red arrows). (G) Western blot analysis of the apoptosis‐related proteins Bax and Bcl‐2. (H–J) ELISA results of IL‐6, IL‐1β, and TNF‐α levels in brain tissue. Data are shown as the mean ± SD (*n* = 6 per group). ***p < 0.001 vs. sham; ###*p* < 0.001 vs. WT + ICH; &&&*p* < 0.001 vs. KO + ICH; $$$*p* < 0.001 vs. WT + ICH + MAT.

Next, *Rage* KO mice were used to confirm the involvement of RAGE signaling in the protective effects of MAT. To verify the genotype of *Rage* KO mice, polymerase chain reaction (PCR) analysis was performed using tail DNA. As shown in Figure S2A, the mixed PCR samples generated unique and clearly distinguishable band patterns on the gels for each RAGE gene and the expected genotypes (WT [+/+] and KO [−/−]) (Figure S2A). Western blot analysis confirmed the absence of RAGE protein expression in the brain tissue of *Rage* KO mice compared to WT controls (Figure S2B). Compared with WT ICH mice, *Rage* KO mice exhibited significantly reduced neurological deficits, as reflected by lower mNSS scores and improved motor coordination in the rotarod test (Figure 6A,B). MAT administration substantially improved these parameters in WT mice, whereas these benefits were markedly diminished in *Rage* KO mice.

Consistent with the behavioral outcomes, RAGE deficiency significantly reduced hematoma volume and Evans blue extravasation, indicating the alleviation of primary hemorrhagic damage and BBB disruption. In *RAGE* KO mice, MAT treatment did not further enhance these effects (Figure 6C,D).

Western blot analysis showed that the ICH‐induced downregulation of tight junction proteins (ZO‐1, Occludin, Claudin‐4) was significantly reversed by either RAGE deletion or MAT treatment in WT mice; however, in *Rage* KO mice, additional MAT administration conferred no additive effect (Figure 6E). Similarly, Nissl staining and apoptotic markers (Bax and Bcl‐2) confirmed that neuronal injury and apoptosis were mitigated by RAGE deficiency and that the efficacy of MAT was largely abolished in *Rage* KO mice (Figure 6F,G).

Furthermore, proinflammatory cytokine levels (IL‐6, IL‐1β, and TNF‐α) were significantly reduced in *Rage* KO mice. MAT significantly attenuated inflammation in WT animals but exhibited minimal benefit in their KO counterparts (Figure 6H–J). These results collectively indicate that the neuroprotective effects of MAT against ICH are largely mediated by inhibition of the RAGE signaling pathway.

## 4 Discussion

ICH remains one of the most devastating forms of stroke, with high morbidity and mortality rates and limited therapeutic options [22]. In the present study, we demonstrate for the first time that MAT, a bioactive quinolizidine alkaloid derived from the TCM *Sophora flavescens* , confers significant neurovascular protection in a murine model of ICH. Our findings reveal that MAT ameliorates hematoma expansion, neurological deficits, BBB disruption, neuronal apoptosis, and neuroinflammation. Mechanistically, these effects are mediated through inhibition of the HMGB1/RAGE signaling axis and suppression of ferroptosis.

An appropriate experimental model is essential for accurately recapitulating the pathophysiological features of ICH. In this study, we selected an autologous blood injection model that closely mimics the clinical scenario of hematoma expansion, mass effect, and secondary brain injury. Consistent with previous reports, our model exhibited substantial hematoma formation and persistent neurological deficits [23]. Importantly, we observed a marked activation of the HMGB1/RAGE axis, as reflected by the upregulation of RAGE, HMGB1, Aβ, PKC, and PLC proteins in perihematomal tissues. Although Aβ is classically associated with Alzheimer's disease, several studies have shown that Aβ expression can also increase following acute brain injury and may act as an endogenous ligand of RAGE, amplifying inflammatory and oxidative stress responses [24, 25]. Therefore, the observed elevation of Aβ in our ICH model likely reflects secondary activation of the HMGB1/RAGE axis rather than amyloidogenic pathology. Previous studies have implicated RAGE activation as a key driver of neuroinflammation and BBB disruption following ICH [12]. These findings not only validate the reliability of our model but also establish a mechanistic basis for targeting the RAGE pathway in subsequent therapeutic interventions.

To select therapeutic targets within the activated pathways, we conducted molecular docking analyses to predict the binding affinity of MAT for key proteins of the RAGE axis. Although MAT has the potential to interact with RAGE, PKC, and PLC, stable hydrogen bond formation was exclusively observed between MAT and Asp81 of RAGE. In contrast, no specific hydrogen‐bonding interactions were identified for PKC or PLC. Hydrogen bonds are critical determinants of ligand–receptor specificity and binding stability and are often correlated with enhanced biological activity [26]. The presence of a stable hydrogen bond between MAT and RAGE suggests a strong and specific interaction, potentially enabling MAT to directly modulate RAGE function. Given the central role of RAGE in orchestrating downstream inflammatory and oxidative responses after ICH, and supported by our docking results, we prioritized RAGE as the primary molecular target of MAT. These findings provide a mechanistic basis for subsequent biological validation and reinforce the therapeutic relevance of directly modulating RAGE signaling, rather than its downstream intermediates.

Following target validation, we systematically evaluated the neuroprotective effects of MAT in a mouse model of autologous blood‐induced ICH. Consistent with its predicted interaction with RAGE, MAT treatment significantly ameliorated the neurological deficits and reduced the hematoma volume in a dose‐dependent manner. Restoration of BBB integrity, as evidenced by decreased EB extravasation and upregulation of tight junction proteins (ZO‐1, Occludin, Claudin‐4), further supports its vascular protective effects. Secondary brain injury following ICH is characterized by widespread neuronal apoptosis and exaggerated inflammatory responses. Previous studies have implicated Bax‐mediated apoptosis and elevated proinflammatory cytokines such as IL‐6, IL‐1β, and TNF‐α in aggravating neuronal loss and neurological deterioration, which are important targets in relieving ICH injury [27, 28]. For example, a recent study demonstrated that CoQ10 administration protects mice against ICH‐induced neurological deficits by relieving neuroinflammation and suppressing neuronal apoptosis [29]. *Lycium barbarum* glycopeptides also exert neuroprotective effects against ICH by alleviating inflammation and apoptosis in the hemorrhagic brain [30]. In line with these findings, our data demonstrated that MAT significantly downregulated Bax while upregulating Bcl‐2 and concurrently suppressed inflammatory cytokine production, thereby mitigating both apoptotic and inflammatory cascades. Importantly, the safety profile of MAT was confirmed by histological analysis and biochemical assays, which showed no detectable hepatic or renal toxicity even at the highest therapeutic dose tested. These results indicate that MAT not only confers neurovascular protection but also possesses a favorable safety margin, reinforcing its translational potential for ICH treatment.

In addition to its anti‐inflammatory and antiapoptotic effects, MAT modulates critical molecular pathways implicated in ferroptosis, a form of regulated cell death driven by iron‐dependent lipid peroxidation. Ferroptosis is increasingly being recognized as a major contributor to neuronal death following ICH [31]. Li et al. found that ferrostatin‐1 (a specific ferroptosis inhibitor) significantly improved the neurological function of mice [32]. Our results showed that ICH induced significant accumulation of Fe^2+^ and dysregulation of ferroptosis‐related proteins, characterized by increased ACSL4 and decreased GPX4 expression, consistent with previous reports [33]. MAT administration reversed these alterations, suggesting the effective suppression of ferroptotic processes.

Mechanistically, ERK1/2 and NF‐κB signaling pathways are well‐established downstream effectors of RAGE activation. Upon ligand binding, RAGE activates intracellular cascades leading to ERK1/2 phosphorylation and NF‐κB p65 nuclear translocation [34, 35], thereby regulating multiple cellular processes, including inflammation. Moreover, the activation of NF‐κB p65 has been shown to contribute to brain damage after ICH [36]. Suppressing NF‐κB p65 activity is associated with improving neurological deficits [37]. Consistently, our study demonstrated that ICH induced robust activation of ERK1/2 and NF‐κB p65 in perihematomal tissue, and that MAT treatment significantly attenuated their phosphorylation. Notably, emerging studies have suggested that ERK1/2 and NF‐κB pathways may also participate in the regulation of ferroptosis under pathological conditions [38, 39]. Given that MAT administration concurrently suppressed ERK1/2 and NF‐κB activation and alleviated ferroptotic injury in our model, it is plausible to hypothesize that MAT mitigates ferroptosis, at least partially, by modulating these downstream signaling pathways. However, the precise mechanistic links between ERK1/2–NF‐κB signaling and ferroptosis regulation in ICH remain to be elucidated and warrant further investigation in future studies.

To further substantiate the dependence of the neuroprotective effects of MAT on RAGE signaling, we used *Rage* KO mice. Consistent with previous reports that RAGE deficiency ameliorates neuroinflammation and tissue damage after central nervous system injury, *Rage* KO mice exhibited markedly attenuated neurological deficits, reduced hematoma volume, preserved BBB integrity, and diminished neuronal apoptosis compared to WT ICH mice. Notably, MAT treatment, which provided significant protection in wild‐type mice, showed minimal additional benefits in *Rage* KO mice.

These results strongly suggest that RAGE is a critical upstream mediator of the therapeutic effects of MAT. In the absence of RAGE, downstream pathological cascades, such as ERK1/2 and NF‐κB activation, ferroptosis progression, and inflammatory cytokine release, are already suppressed, thereby diminishing the observable impact of MAT intervention. This genetic validation underscores the centrality of RAGE in mediating secondary brain injury after ICH and confirms that the direct or indirect modulation of RAGE represents a viable therapeutic strategy.

Although our findings support RAGE as the primary molecular target of MAT, it should be noted that MAT has also been reported to influence other signaling cascades involved in neuroinflammation and oxidative stress, including the NF‐κB, MAPK, and Nrf2 pathways, in different models of neurological injury. Thus, we cannot completely exclude the possibility that MAT exerts additional off‐target or indirect effects in addition to HMGB1/RAGE modulation. Future studies integrating proteomic screening or pharmacological inhibition approaches will be valuable for delineating the broader signaling landscape regulated by MAT.

Collectively, our findings demonstrate, for the first time, that MAT confers neurovascular protection in ICH by directly targeting the HMGB1/RAGE signaling axis and inhibiting ferroptosis. Unlike previous studies that have focused solely on downstream inflammatory mediators or oxidative pathways, our work highlights the therapeutic potential of modulating upstream regulators to achieve broader neuroprotective effects. Furthermore, the integration of molecular docking analysis with biological validation provides mechanistic insights into the ligand–target interactions between MAT and RAGE.

Nevertheless, this study has several limitations. The exact structural basis of the MAT–RAGE interaction requires further validation using crystallographic or biophysical approaches. Additionally, although ferroptosis markers were assessed, direct visualization (e.g., transmission electron microscopy) was not performed. Further studies are required to explore the long‐term efficacy of MAT and its synergy with other therapeutic strategies.

In conclusion, our findings demonstrate that MAT exerts neuroprotective effects in ICH by targeting the HMGB1/RAGE axis and ferroptosis, thereby offering a novel therapeutic avenue for the treatment of hemorrhagic stroke.

## Funding

This research was supported by the Medical and Health Research Project of Zhejiang Province (2025KY1258) and the National Natural Science Foundation of China (82171366).

## Conflicts of Interest

The authors declare no conflicts of interest.

## Supporting information


**Figure S1:** Evaluation of the safety profile of MAT in ICH mice. (A and B) Representative H&E‐stained sections of liver and kidney tissues from each group. (C–F) Quantitative analysis of the serum biochemical parameters AST, ALT, BUN, and creatinine. No histological or biochemical abnormalities were observed in the MAT‐treated mice. Data are expressed as the mean ± SD (*n* = 6 per group).

**Figure S2:** Verification of the knockout status of *Rage* KO mice. (A) PCR genotyping results of *Rage* WT and KO mice. (B) Western blot validation of RAGE protein expression.

## Data Availability

The data that support the findings of this study are available from the corresponding author and first author upon reasonable request.
