# Supplementary figures and images for: Matrine Attenuates Neurological Deficits and Neuroinflammation by Inhibiting the HMGB1/RAGE Axis and Ferroptosis in Intracerebral Hemorrhage Mice
Source: Kaohsiung J Med Sci. 2025 Dec 16;42(7):e70157. doi: 10.1002/kjm2.70157 (PMC13344244; doi:10.1002/kjm2.70157)

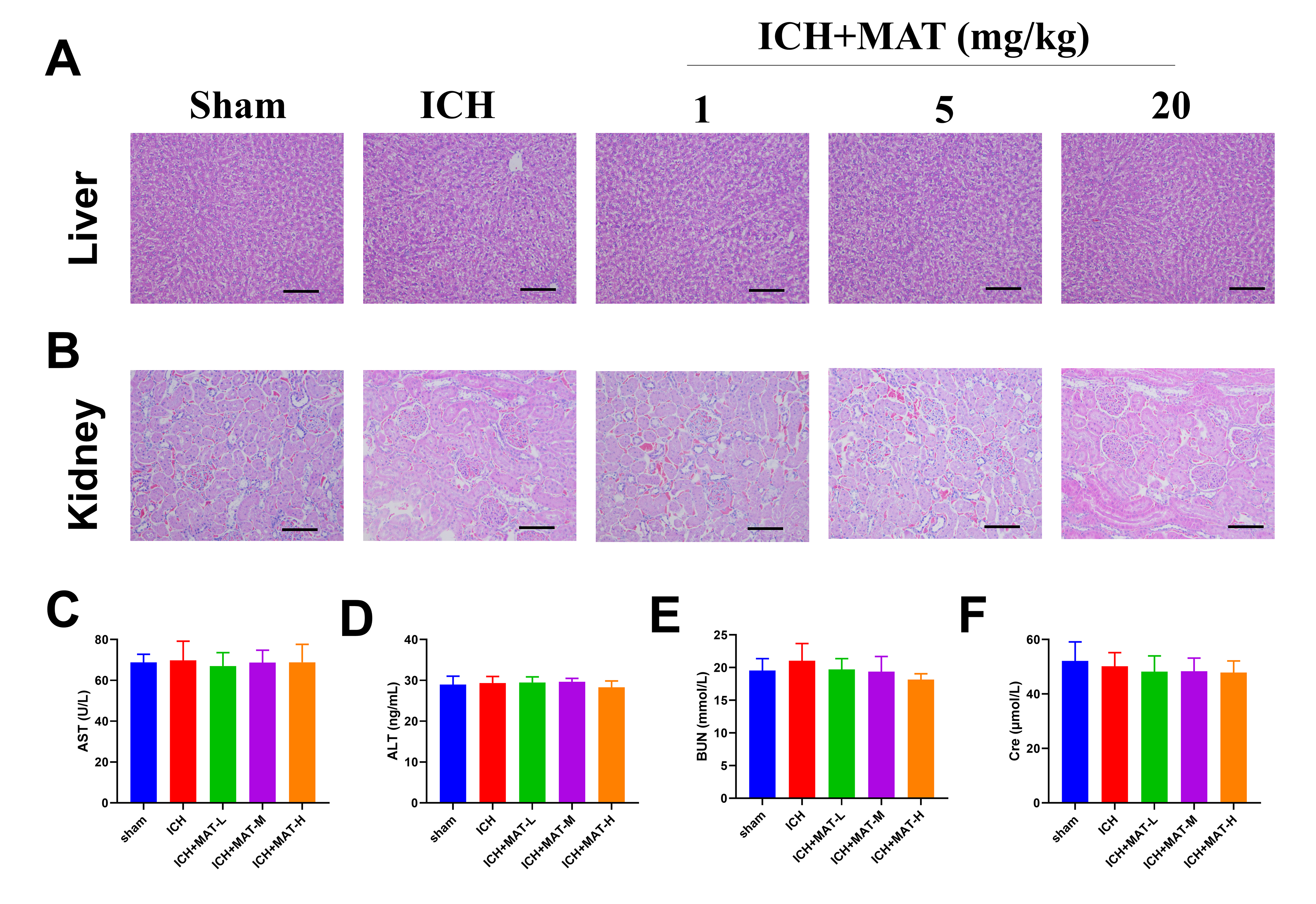

Supplement: Supplementary file 1 — Figure S1: Evaluation of the safety profile of MAT in ICH mice. (A and B) Representative H&E‐stained sections of liver and kidney tissues from each group. (C–F) Quantitative analysis of the serum biochemical parameters AST, ALT, BUN, and creatinine. No histological or biochemical abnormalities were observed in the MAT‐treated mice. Data are expressed as the mean ± SD (n = 6 per group). [file KJM2-42-e70157-s001.tif]

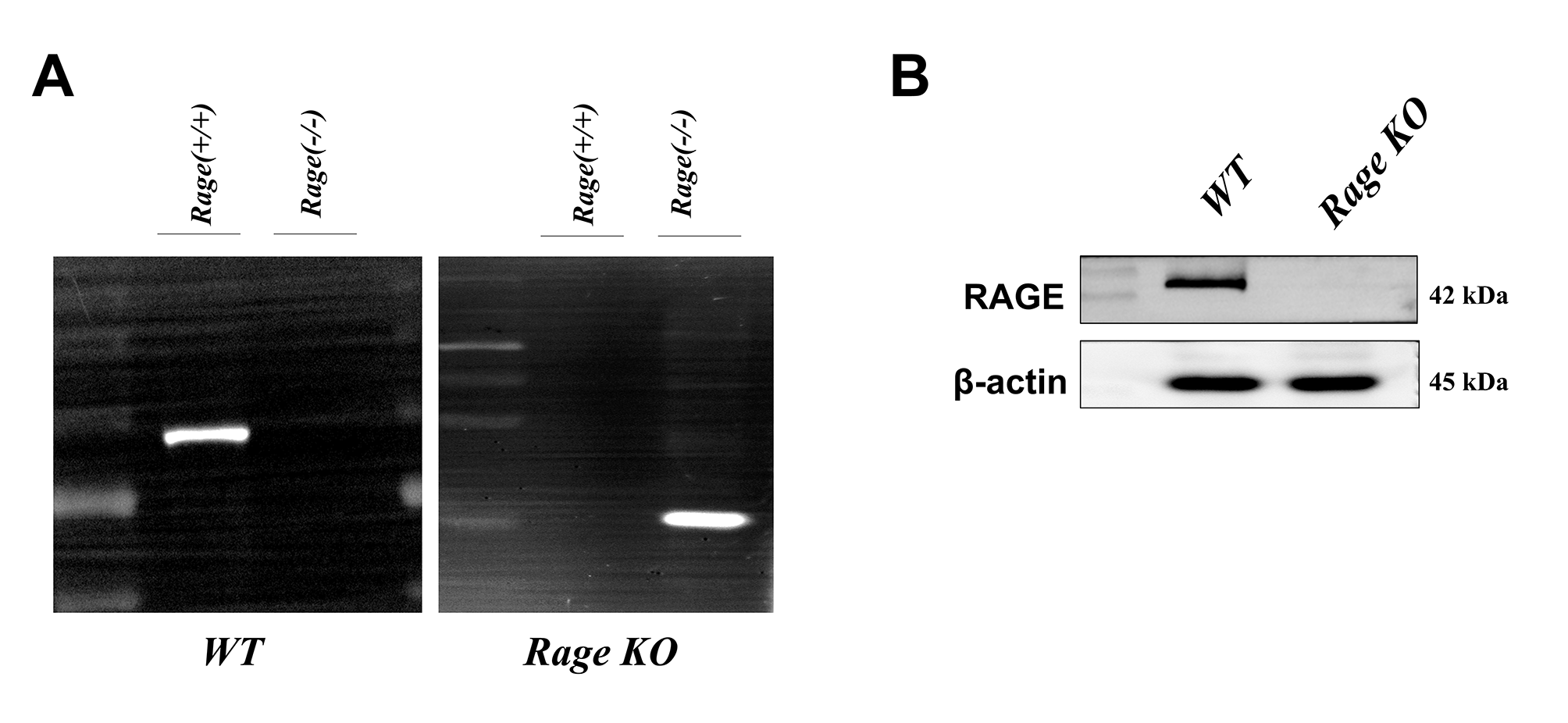

Supplement: Supplementary file 2 — Figure S2: Verification of the knockout status of Rage KO mice. (A) PCR genotyping results of Rage WT and KO mice. (B) Western blot validation of RAGE protein expression. [file KJM2-42-e70157-s002.tif]
